# Phrenic nerve stimulation for central sleep apnea: a single institution experience

**DOI:** 10.1007/s11325-024-03125-x

**Published:** 2024-07-31

**Authors:** Julie Mease, Ralph Augostini, Scott McKane, Meena Khan

**Affiliations:** 1 Division of Cardiology, Department of Internal Medicine, The Ohio State University Wexner Medical Center, Columbus, OH USA; 2 Division of Pulmonary Critical Care and Sleep Medicine, Department of Internal Medicine, Ohio State University Medical Center, Columbus, OH USA; 3 Biostatistics and Data Management, Zoll Respicardia, Minnetonka, MN USA

**Keywords:** Central sleep apnea, Phrenic nerve stimulation, Central apnea index, Epworth sleepiness scale, Functional outcomes of sleep questionnaire

## Abstract

**Purpose:**

Phrenic nerve stimulation (PNS) was approved by the Food and Drug Administration (FDA) to treat moderate to severe central sleep apnea. We report here, results of a retrospective study regarding our institutional outcomes at one year. In this study we evaluated the change in the apnea hypopnea index, epworth sleepiness score, and functional outcomes of sleep score at one year post implant.

**Methods:**

This is a retrospective analysis of patients ≥ 18 years of age who had PNS implanted for moderate to severe CSA at the Ohio State University Wexner Medical Center apnea between Feb 1, 2018 to July 1, 2021. Sleep disordered breathing parameters and objective sleepiness as measured by the Epworth Sleepiness Scale (ESS) scores, and Functional Outcomes of Sleep Questionnaire (FOSQ) scores were assessed at baseline and one-year post-implant.

**Results:**

Twenty-two patients were implanted with PNS at OSU between February 1, 2018 and May, 31, 2022. The AHI showed a statistically significant decrease from a median of 40 events/hour at baseline to 18 at follow-up (p-value = 0.003). The CAI decreased from 16 events/hour to 2 events/hour (p-value of 0.001). The obstructive apnea index, mixed apnea index, and hypopnea index did not significantly change. The ESS scores had a statistically significant improvement from a median score of 12 to 9 (p-value = 0.028). While the FOSQ showed a trend to improvement from 15.0 to 17.8, it was not statistically significant (p-value of 0.086).

**Conclusion:**

Our study found that PNS therapy for moderate to severe CSA improves overall AHI and CAI. Objective sleepiness as measured by the ESS also improved at one-year post implant.

## Introduction

Central sleep apnea (CSA) is a condition where there is a reduction or cessation of airflow associated with lack of respiratory muscle effort [1]. CSA has been associated with difficulty sleeping, unrefreshing sleep, and excessive daytime sleepiness. Moderate to severe CSA (central apnea index (CAI) > = 15 central apneas/hour of sleep) has been associated with conditions such as congestive heart failure, altered heart rate variability, and arrhythmias such as nonsustained ventricular tachycardia and atrial fibrillation [2–6] .

Treatment of CSA has primarily been positive airway pressure (PAP) therapy. Other treatment considerations include optimizing heart function in those with heart failure, supplemental oxygen, and medications such as acetazolamide. Phrenic nerve stimulation (PNS) was approved by the Food and Drug Administration (FDA) in 2017 for moderate to severe central sleep apnea in adults. PNS is transvenous implantable device that stimulates the phrenic nerve unilaterally through a nearby vein. The stimulation causes contraction of the hemidiaphragm. The **rem**edē System (a PNS device) was studied in a multicenter trial of 151 patients. The study randomized subjects with moderate to severe CSA (apnea hypopnea index (AHI) of > = 20) to PNS therapy or a control group. The subjects also had to have > 50% central apnea and > = 30 central apneas events in the diagnostic study. The obstructive apnea index also had to be < = 20% of the total AHI. At 12 months, 67% of patient in the treatment group had a > = 50% reduction in AHI. The CAI significantly improved while the obstructive and hypopnea index did not statistically change. 60% of patients felt marked or moderate subjective improvement by patient global assessment scale [7]. There improvements continued at 5 years post implant [8]. We publish here our institutional experience with those implanted with PNS therapy for CSA.

## Methods

This is a retrospective study evaluating outcomes of adults (age > = 18 years of age) who had PNS implanted and managed to treat moderate to severe central sleep apnea between Feb 1, 2018 to July 1, 2021 at The Ohio State University Wexner Medical Center. Patient data was gathered at baseline (prior to implant) and a follow-up visit closest to one year after implant to assess efficacy, symptom improvement, and safety. This retrospective study was approved by The Ohio State University Institutional Review Board (IRB).

Data collection included baseline characteristics, sleep study results, Epworth Sleepiness Scale (ESS), and Functional Outcomes of Sleep Questionnaire (FOSQ). ESS is a validated 24-point subjective scale used to measure daytime sleepiness with values above 10 suggesting excessive daytime sleepiness [9]. Functional Outcomes of Sleep Questionnaire (FOSQ) score, is a scale from 5 to 20 with lower scores indicating impaired sleep-related quality of life [10]. On-therapy sleep study, ESS, and FOSQ results closest to 1-year post-implant were extracted to assess impact of PNS therapy. Safety was also assessed through 1 year post-implant.

Baseline sleep study testing in all but one patient was from an in-lab polysomnogram. One baseline study was a home sleep apnea test result and one of the in lab polysmonograms was a split night study where the baseline sleep disordered breathing data was taken from the diagnostic portion. One year follow up results were mix of in lab polysmnogram and home sleep apnea test. This is due to home sleep apnea tests being ordered on patients at one year who refuse to have in lab polysomnogram completed.

### Statistical analysis

Continuous variables were summarized by median and 1st (Q1) and 3rd (Q3) quartile due to the skewed distribution of sleep study parameters. Categorical variables are summarized by the percentage of patients in each category. For sleep metrics, ESS and FOSQ, baseline and follow-up visit results and paired change from baseline (follow-up result minus baseline) are presented. Sleep study parameters requiring an electroencephalogram signal, such as sleep stages and arousal index, are not available for the home sleep tests and therefore were excluded from the on-therapy analyses due to small sample size.

Although the study was not powered and the sample size was small, p-values for change from baseline were calculated using the non-parametric Wilcoxon Signed-Rank test. No adjustment for multiplicity was performed and a nominal p-value < 0.05 was considered statistically significant.

Since this was a retrospective study, there is missing or incomplete data for some of the patients. No imputation for missing data was performed. Analyses were performed with SAS version 9.4 (Cary, NC).

## Results

Twenty-two patients were implanted with the phrenic nerve stimulator at OSU between February 1, 2018 and May, 31, 2022. The average duration from implant to follow up data closest to one year was 13 months. Three patients were lost to follow up. There is baseline data on AHI for all 22 patients, ESS for 17 patients, and FOSQ for 13 patients. The reason for absent ESS and FOSQ data at baseline was due to patients not being seen in a sleep medicine clinic visit before implant and having the electronic medical record reviewed by a sleep medicine physician instead. In terms of one year data, there is AHI for 19 patients, ESS for 16 patients, and FOSQ for 14 patients, although paired data for only 13 with ESS and 10 with FSS at both visits. There is missing ESS on 3 patients and FOSQ on 5 patients who completed the sleep study follow up but did not complete follow up sleep medicine visits.

### Baseline characteristics

Baseline characteristics are reported in Table 1. The median age was 73 [1st and 3rd quartile: 65, 77] years, the median body mass index (BMI) was 25 [23, 30] kg/m^2^, and 82% were male. The median ESS and FOSQ scores were 12 [11, 14] and 15 [13, 18.5], respectively. In terms of co-morbid cardiovascular disease: 36% had heart failure, 36% atrial fibrillation, 27% coronary artery disease, 27% valve disease, and 36% had an implantable electronic cardiac device (5 CRT-D and 3 pacemaker). There were no patients with opiate induced central sleep apnea in this cohort.

**Table 1:** Baseline characteristics

Baseline Characteristics	Implanted patients^1^ (N = 22)
Male	18 (82%)
White race	19 (86%)
Age (years)	73 [65, 77]
BMI (kg/m^2^)	25 [23, 30]
Epworth Sleepiness Scale (points)	12 [11, 14] (*n* = 17)
Functional Outcomes of Sleep Questionnaire	15 [13, 18.5] (*n* = 13)
Heart failure	8 (36%)
New York Heart Classification (I/II/III/IV)	0% / 6 (27%) / 2 (9%) / 0%
Left ventricular ejection fraction (%)	59 [45,63] (*n* = 18)
Coronary artery disease	6 (27%)
Valve disease	6 (27%)
Atrial fibrillation	8 (36%)
Paroxysmal	2 (9%)
Persistent	6 (27%)
Non-sustained ventricular tachycardia	1 (5%)
CRT-D	5 (23%)
Pacemaker	3 (14%)
Neuromuscular disease	1 (5%)
Opiate medication	0 (0%)

^1^Continuous variables reported as median [1st quartile, 3rd quartile] and categorical displayed as n (percent)

n- number

%-percentage

Kg/m2 – kilogram per meters squared

CRT-D-cardiac resynchronization therapy- defibrillator

The median baseline AHI was 40 [23, 47] events/hour of sleep indicating severe CSA, with the majority of events being central apneas (central apnea index [CAI] 16 [11, 31]) followed by hypopneas (hypopnea index [HI] 14 [4, 23]). Hypopneas were not designated as central or obstructive. The obstructive apnea index and mixed apnea index were low at 2 [0, 4] and 0 [0, 2], respectively. The median 4% oxygen desaturation index (ODI) was 32 [19,42]. Patients experienced a median of 26 [17, 36] arousal events/hour of sleep.

### One year follow up data

**Fig. 1:**
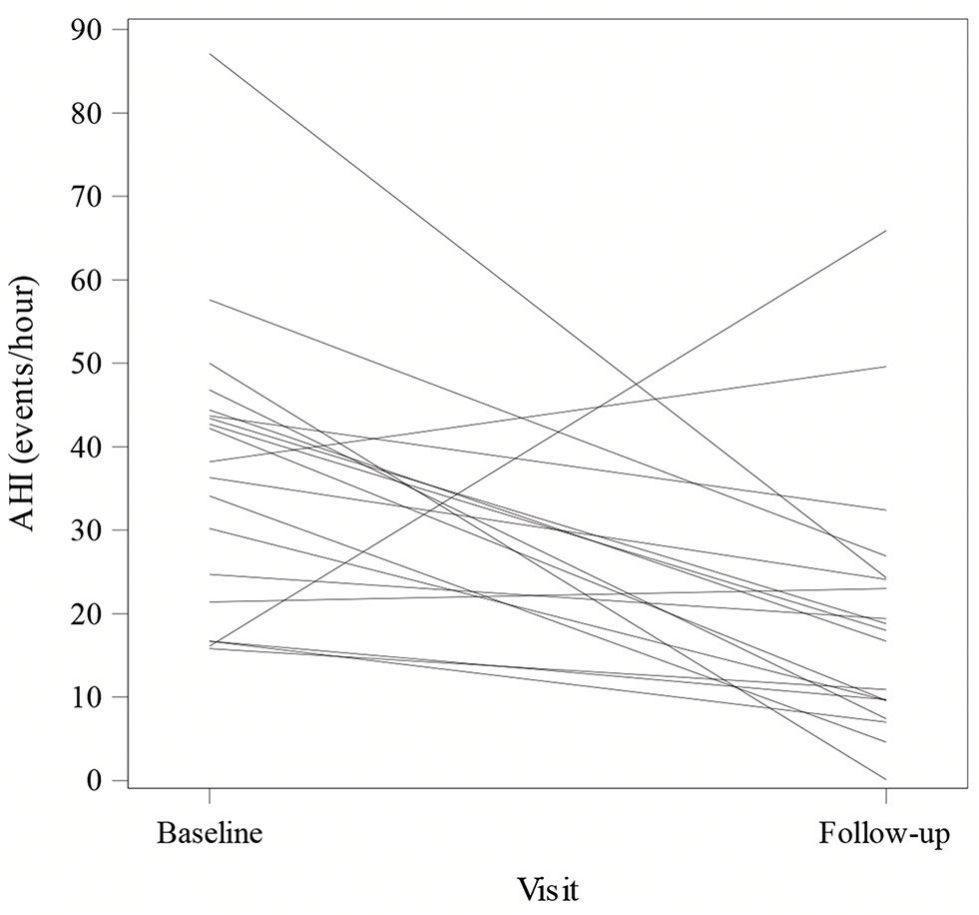
Line plot change in the apnea hypopnea index (AHI). The change in the apnea hypopnea index (AHI) from baseline to one year follow up is displayed for each patient

**Fig. 2:**
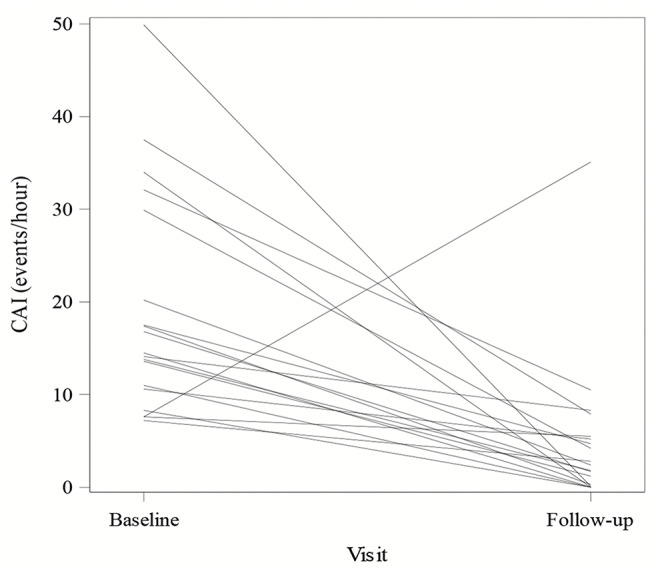
Line plot of the change in the central apnea index (CAI). The change in central apnea index (CAI) from baseline to one year follow up is displayed for each patient

The AHI decreased from a median of 40 [23, 47] events/ hour at baseline to 18 [10, 24] at follow-up, yielding a median change of -21 [-31, -5] events/hour which is a statistically significant improvement (p-value = 0.003). The CAI decreased from 16 [11, 31] events/hour to 2 [0, 6] events/hour which was also a statistically significant decrease (p-value < 0.001); Figs. 1 and 2 displays the change in AHI and CAI for each subject. Of note, there is one patient whose AHI worsened at one year due to increase in obstructive apneas. The central apneas did improve with PNS therapy. There is also a second patient whose AHI and CAI worsened at follow up testing. This patient was admitted to the hospital days after his post implant sleep study and found to have a reduced ejection fraction and subsequently died after cardiac arrest. The ODI improved from 32 [19, 42] to 15 [10, 22] with a median change of -17 [-24, -2] (p-value of 0.033). The obstructive apnea index (median 2 events/hour) and mixed apnea index (median 0 events/hour) were low at baseline and did not show significant changes at one year (both with p-value > 0.3). The hypopnea index, which was not differentiated as central versus obstructive, was 14 [4, 23] events/hour and decreased to 8 [2, 16] at one year which was not a significant change (median paired change from baseline was − 1 [-12, 7] event/hour with p-value > 0.3). Sleep disordered breathing metrics at baseline and follow-up are displayed in Table 2.

**Table 2:** Change in respiratory parameters from baseline to one year post -implant

Endpoint	Baseline^1^ (n = 22)	Follow-up (n = 19)		
		Result	Change from baseline	2-sided p-value^2^
Apnea Hypopnea Index (events/hour)	40 [23, 47]	18 [10, 24]	-21 [-31, -5]	0.003
Central Apnea Index (events/hour)	16 [11, 31]	2 [0, 6]	-13 [-22, -6]	< 0.001
Obstructive Apnea Index (events/hour)	2 [0, 4]	2 [0, 10]	0 [-2, 8]	0.404
Mixed Apnea Index (events/hour)	0 [0, 2]	0 [0, 1]	0 [-2, 0]	0.367
Hypopnea Index (events/hour)	14 [4, 23]	8 [2, 16]	-1 [-12, 7]	0.374
Oxygen Desaturation Index 4% (ODI4) (events/hour)	32 [19, 42] (*n* = 16)	15 [10, 22] (*n* = 18)	-17 [-24, -2] (*n* = 14)	0.033

^1^Median [Q1, Q3]

^2^P-value from Wilcoxon Signed-Rank test

n = number

Symptoms of excessive daytime sleepiness were measured using ESS and FOSQ. The baseline ESS median value was 12 [11, 14] and improved by a median of 3.0 points at follow-up to 9 [5.0, 12.5] (p-value = 0.028). A clinically meaningful 2-point improvement was observed in 69% of the 13 patients with paired data. The FOSQ, improved from 15.0 [13.0, 18.5] to 17.8 [17.0, 19.5] at follow-up, with paired change from baseline of 3.0 [-1.5, 5.0] (p-value = 0.086); Five of the 10 subjects with data at both visits had a clinically meaningful improvement of 1.8 or more [10]. (Table 3).

**Table 3:** Change in quality of life metrics from baseline to one year post-implant

Endpoint	Baseline^1^	Follow-up (n = 19)		
		Result	Change from baseline	2-sided *p*-value^2^
Epworth Sleepiness Scale (points)^a^	12.0 [11.0, 14.0] (*n* = 17)	9.0 [5.0, 12.5] (*n* = 16)	-3.0 [-6.0, -1.0] (*n* = 13)	0.028
Functional Outcomes of Sleep Questionnaire (points)	15.0 [13.0, 18.5] (*n* = 13)	17.8 [17.0, 19.5] (*n* = 14)	3.0 [-1.5, 5.0] (*n* = 10)	0.086

^1^ Median [Q1, Q3]

^2^ P-value from Wilcoxon Signed-Rank test

n = number

Since an HSAT may underestimate the AHI by including awake time in the index calculations, a sensitivity analysis of the subset of 11 patients who had a PSG at both baseline and follow-up was performed (Table 4). In this analysis, the AHI improved from a median of 42 [21, 47] to 11 [7, 23] at one year (p-value = 0.003). The CAI improved from a median of 17 [14, 30] to 2 [0, 6] (p-value < 0.001) and hypopnea index improved from a median of 11[4, 22] to 5 [2, 16] (p-value = 0.103). These results are similar to the overall study results.

**Table 4:** Change in respiratory parameters from baseline to one year post-implant using polysomnography only

Endpoint	Baseline^1^ (n = 11)	Follow-up (n = 11)		
		Result	Change from baseline	2-sided p-value^2^
Apnea Hypopnea Index (events/hour)	42 [21, 47]	11 [7, 23]	-21 [-39, -5]	0.003
Central Apnea Index (events/hour)	17 [14, 30]	2 [0, 6]	-15 [-26, -8]	< 0.001
Obstructive Apnea Index (events/hour)	0 [0, 2]	0 [0, 2]	0 [-1, 2]	0.461
Mixed Apnea Index (events/hour)	0 [0, 2]	0 [0, 1]	0 [-2, 0]	0.630
Hypopnea Index (events/hour)	11 [4, 22]	5 [2, 16]	-5 [-12, 1]	0.103

^1^ Median [Q1, Q3]

^2^ P-value from Wilcoxon Signed-Rank test

n = number

We did look at ejection fraction. Only 8 subjects in this cohort had a diagnosis of heart failure. When looking at the entire cohort, the median left ventricular ejection fraction was 59% [45, 63] (n = 18) at baseline and 45% [25, 65] (n = 10) at follow-up. However most subjects with a normal left ventricular ejection fraction (LVEF) at baseline did not have a follow-up echocardiogram. We have paired data on 9 subjects with data at baseline and follow-up, LVEF increased by a median of 5 [0, 8] percentage points. Of the 9 subjects with LVEF data at baseline and follow-up, median LVEF was 45 [25, 60] at baseline and 50 [30, 65] at follow-up, yielding a 5 [0, 8] percentage point increase (p-value = 0.133). While this change is not significant, the number of subjects with this data was too small to draw meaningful conclusions.

## Safety

Two of the 22 patients experienced a hematoma after the implant procedure, and both were treated without sequela. Stimulation discomfort initially with therapy was common, but could be resolved with adjustment of the settings in all patients. One patient needed the stimulation lead to be replaced since it would not remain in a stable position to allow consistent therapy delivery. After lead replacement, the patient was able to resume therapy without issues. None of the patients in this cohort had device removal.

## Discussion

This retrospective study reports our institutional experience with PNS for CSA at one year post implant. We found a significant reduction in AHI and CAI. We did not find a significant change in the hypopnea index, obstructive apnea index, or mixed apnea index. There was one patient who had worsening AHI and CAI with PNS therapy. This patient died a few days after their follow-up sleep study likely due to cardiac issues unrelated to PNS therapy. The worsening sleep disordered breathing may be reflective of worsening cardiac status as opposed to failure of PNS therapy. There was significant improvement in sleepiness as measured by ESS. There was improvement in FOSQ though it did not reach the threshold for statistical significance, however the test was underpowered to detect a change. Our findings are similar to that of the pivotal trial [7, 8]. There are also meta-analyses published that show improvement in AHI, CAI, and arousal index in those implanted with PNS for CSA [11, 12].

Phrenic nerve stimulation is an effective treatment for CSA via improvement of central apneas. Hypopneas did decrease but not to a statistically significant degree. Hypopneas were not scored as obstructive or central. It is possible that there were central hypopneas that improved but may still remain as part of the residual AHI at one year. It is also likely that many of the hypopneas were obstructive and would not be expected to improve with PNS therapy.

Further studies would be helpful to specifically identify the types of residual events that occur (i.e. central versus obstructive hypopneas) and outcomes with combination therapies with PNS such as positional therapy [13] or PAP therapy for residual obstructive events to further improve the treatment AHI and patient symptoms.

There are limitations to our study. This is a small cohort of patients with missing data points at baseline and one year for some of the patients. Three patients were lost to follow up at one year as well. This cohort was also too small to draw any conclusions regarding CSA treatment with PNS in terms of morbidity, ejection fraction, or re-admission rates to the hospital. Also, the hypopneas were not scored as obstructive or central at baseline or post-treatment. This precludes our ability to draw conclusions on the effect of PNS therapy on central hypopneas.

## Conclusions

Phrenic nerve stimulation is an effective treatment of central sleep apnea as measured by improvement of overall AHI, CAI, and ODI. Excessive daytime sleepiness also improves with therapy. Future studies are needed to evaluate for associations in terms of possible effects on ejection fraction, hospital re-admission rates, and other co-morbidities. Prospective directions of this therapy include scoring hypopneas as obstructive or central based on the American Academy of Sleep Medicine Scoring Manual in order to further address how effective PNS therapy is managing these events. Also, more data needs to be collected regarding efficacy of the therapy in those with opiate induced central sleep apnea as well as combination therapy options for those with obstructive and central sleep apnea who do not tolerate PAP therapy.

## Data Availability

The data that support the findings of this study are not openly available due to reasons of sensitivity and are available from the corresponding author upon reasonable request. Data are located in controlled access data storage in an Ohio State University protected computer drive.
